# Valor Prognóstico da Classificação Angiográfica de Hata para Desfechos em Longo Prazo na Arterite de Takayasu: Um Estudo Retrospectivo de Centro Único

**DOI:** 10.36660/abc.20250786

**Published:** 2026-06-15

**Authors:** Anisio Uchoa Leite Santana, Samuel Katsuyuki Shinjo

**Affiliations:** 1 Divisão de Reumatologia Faculdade de Medicina Universidade de São Paulo São Paulo SP Brasil Divisão de Reumatologia, Faculdade de Medicina da Universidade de São Paulo (FMUSP), São Paulo, SP – Brasil

**Keywords:** Arterite de Takayasu, Prognóstico, Vasculite Sistêmica

## Abstract

**Fundamento:**

A utilidade prognóstica da classificação angiográfica de Hata para desfechos em longo prazo na arterite de Takayasu (AT) permanece incerta.

**Objetivos:**

Avaliar o valor prognóstico da classificação de Hata na predição de eventos cardiovasculares adversos maiores (ECAM) e da necessidade de intervenções vasculares em pacientes com AT.

**Métodos:**

Este estudo de coorte retrospectivo, de centro único, incluiu pacientes diagnosticados com AT entre 2000 e 2025. Análises de regressão logística multivariada foram realizadas para identificar preditores independentes, considerando-se significância estatística quando p < 0,05.

**Resultados:**

Dos 203 pacientes inicialmente identificados, 18 foram excluídos. A coorte final foi composta por 185 pacientes, dos quais 80,0% eram do sexo feminino, com mediana de idade ao diagnóstico de 29 anos. O Tipo V foi o padrão angiográfico predominante (64,9%). Durante um seguimento mediano de 10,8 anos, ECAM ocorreram em 35,7% dos pacientes, enquanto 37,3% necessitaram de intervenção vascular. A classificação de Hata não apresentou associação com a ocorrência de ECAM. Os preditores independentes de ECAM incluíram sexo feminino, associado a efeito protetor (*odds ratio* [OR], 0,38; intervalo de confiança de 95% [IC 95%], 0,17-0,86; p = 0,021), e maior intervalo entre o início dos sintomas e o diagnóstico, associado a aumento do risco (OR, 1,10; IC 95%, 1,03-1,18; p = 0,006). Em contraste, a classificação de Hata foi preditora independente da necessidade de intervenção vascular. O Tipo I de Hata apresentou forte associação protetora em comparação ao Tipo V (OR, 0,10; IC 95%, 0,01-0,53; p = 0,029). Além disso, insuficiência aórtica basal associou-se independentemente a maior probabilidade de intervenção (OR, 2,54; IC 95%, 1,18-5,60; p = 0,018) (Figura Central).

**Conclusões:**

A classificação de Hata demonstrou ser um preditor robusto de necessidade de intervenção vascular, mas não de ECAM, em pacientes com AT. Maior extensão anatômica da doença (Tipo V) e insuficiência aórtica basal associaram-se a maior risco de procedimentos vasculares, enquanto o risco de ECAM esteve relacionado a atraso diagnóstico e sexo masculino. A dissociação observada em longo prazo entre elevadas taxas de remissão clínica (92,4%) e progressão contínua da doença reforça a importância do acompanhamento baseado em métodos de imagem e destaca a necessidade de estudos prospectivos multicêntricos para validação desses achados.

## Graphical abstract

**Figura Central:**
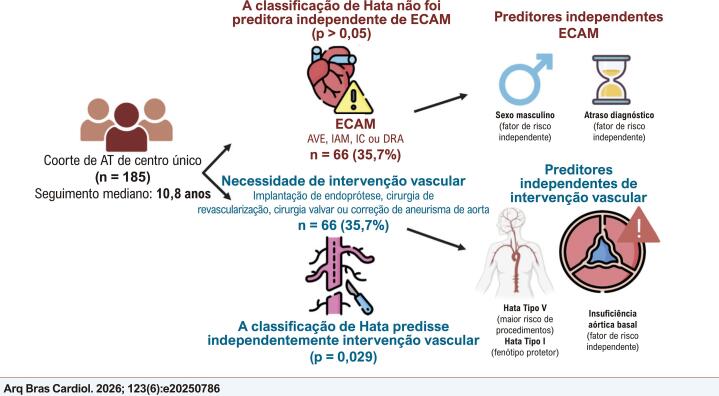
Valor Prognóstico da Classificação Angiográfica de Hata para Desfechos em Longo Prazo na Arterite de Takayasu: Um Estudo Retrospectivo de Centro Único

*AT: arterite de Takayasu; AVE: acidente vascular encefálico; DRA: dilatação da raiz da aorta; ECAM: eventos cardiovasculares adversos maiores; IAM: infarto agudo do miocárdio; IC: insuficiência cardíaca.*

## Introdução

A arterite de Takayasu (AT) é uma vasculite granulomatosa crônica rara que afeta predominantemente artérias de grande calibre, particularmente a aorta e seus principais ramos.^1-3^ A doença ocorre principalmente em mulheres jovens, geralmente durante a segunda e a terceira décadas de vida. O processo inflamatório subjacente promove espessamento e fibrose da parede arterial, levando, em última instância, ao desenvolvimento de lesões estenóticas, oclusivas ou aneurismáticas.^4^ Essas alterações estruturais representam importantes determinantes de morbidade e mortalidade e frequentemente culminam em complicações isquêmicas graves, incluindo acidente vascular encefálico (AVE), infarto agudo do miocárdio (IAM), hipertensão renovascular (HRV) e insuficiência cardíaca (IC).^5,6^

A expressiva heterogeneidade clínica e a variabilidade da evolução da doença tornam a predição de desfechos em longo prazo na AT um desafio importante na prática clínica.^5^ Nesse contexto, diferentes sistemas de classificação angiográfica foram desenvolvidos com o objetivo de padronizar a caracterização anatômica do acometimento arterial. Entre eles, a classificação proposta por Hata et al.^7^ consolidou-se como o método mais amplamente utilizado, organizando a AT em seis padrões angiográficos distintos (Tipos I, IIa, IIb, III, IV e V), definidos de acordo com a distribuição anatômica do acometimento vascular. Essa classificação tornou-se um importante referencial para caracterização de populações de pacientes e comparação de resultados em estudos clínicos.

Evidências anteriores demonstraram associação entre os padrões angiográficos de Hata e diferentes manifestações clínicas da doença. Uma metanálise recente identificou o Tipo V, caracterizado pela forma angiográfica mais extensa de acometimento vascular, como o subtipo mais prevalente e também aquele associado à maior carga global de sintomas.^8^ Estudos adicionais demonstraram relações entre padrões angiográficos específicos e complicações clínicas distintas, incluindo maior risco de eventos cerebrovasculares em pacientes classificados como Tipo IIa.^9^ Entretanto, a determinação do perfil prognóstico da AT permanece complexa. Estudos de coorte de grande porte identificaram múltiplos fatores independentes associados a desfechos desfavoráveis, incluindo progressão contínua da doença, acometimento da aorta torácica, retinopatia e elevação de biomarcadores inflamatórios.^5,6^ Apesar desses avanços, o papel prognóstico específico da classificação angiográfica de Hata ainda permanece incompletamente estabelecido.

Embora amplamente empregada para caracterização anatômica da AT, permanece uma importante lacuna de conhecimento quanto às implicações prognósticas diretas da classificação angiográfica de Hata sobre desfechos clínicos em longo prazo. Dessa forma, o presente estudo teve como objetivo avaliar o valor prognóstico da classificação de Hata por meio da análise das associações entre padrões angiográficos e desfechos clínicos em longo prazo em uma grande coorte retrospectiva de centro único composta por pacientes com AT. Adicionalmente, buscou-se investigar se subtipos específicos da classificação de Hata podem contribuir para a estratificação do risco de eventos cardiovasculares adversos maiores (ECAM) e necessidade de intervenções vasculares.

## Métodos

### Delineamento do estudo

Este estudo de coorte retrospectivo, de centro único, incluiu pacientes com AT acompanhados na unidade de vasculites de nosso centro terciário entre janeiro de 2000 e outubro de 2025. O estudo foi conduzido em conformidade com os princípios da Declaração de Helsinque e aprovado pelo Comitê de Ética em Pesquisa com Seres Humanos da instituição.

Os participantes elegíveis deveriam apresentar diagnóstico confirmado de AT de acordo com os critérios classificatórios do American College of Rheumatology (ACR) de 1990^10^ e os critérios classificatórios da European Alliance of Associations for Rheumatology/ACR de 2022.^11^ Pacientes com dados incompletos ou indisponíveis referentes à classificação angiográfica foram excluídos.

O relato do estudo seguiu as recomendações da diretriz STrengthening the Reporting of OBservational Studies in Epidemiology.

### Coleta de dados

Os dados foram extraídos sistematicamente dos prontuários eletrônicos por meio de protocolo padronizado. As variáveis incluíram características demográficas e basais, como idade ao diagnóstico, sexo, etnia e duração do seguimento; classificação angiográfica segundo Hata et al.^7^ (Tipos I, IIa, IIb, III, IV e V); e variáveis relacionadas ao tratamento, incluindo medicamentos utilizados durante o acompanhamento, como glicocorticoides, agentes imunossupressores, agentes imunomoduladores, terapias biológicas, antiagregantes plaquetários e estatinas.

Também foram avaliados desfechos e complicações em longo prazo, incluindo mortalidade, eventos cardiovasculares adversos maiores (ECAM) (IAM, IC, eventos cerebrovasculares e dissecção de aorta) e necessidade de intervenções vasculares. Além disso, foram registradas comorbidades e complicações clínicas, incluindo hipertensão arterial sistêmica (HAS), HRV, insuficiência aórtica, diabetes melito, dislipidemia e hipertensão pulmonar.

Foram definidos dois desfechos prognósticos compostos primários: i) ECAM, composto por AVE, IAM, IC e dissecção de aorta; e ii) intervenção vascular, definida pela necessidade de implantação de endoprótese, cirurgia de revascularização, cirurgia valvar ou correção de aneurisma de aorta.

O estado da doença na última consulta de seguimento foi categorizado como doença ativa ou remissão. A atividade da doença foi avaliada pelo Indian Takayasu Clinical Activity Score (ITAS2010),^12^ sendo considerados indicativos de doença ativa escores ≥ 2.

### Análise estatística

Estatísticas descritivas foram utilizadas para caracterizar a coorte do estudo. As variáveis contínuas não apresentaram distribuição normal segundo o teste de Shapiro-Wilk e, portanto, foram apresentadas como mediana e intervalo interquartil (IIQ; percentis 25-75). Variáveis categóricas foram descritas como frequências absolutas e porcentagens.

As características basais entre os grupos da classificação de Hata foram comparadas utilizando o teste de Kruskal-Wallis para variáveis contínuas e o teste do qui-quadrado ou o teste exato de Fisher para variáveis categóricas, sem realização de análises *post hoc* pareadas.

Para avaliar o valor prognóstico da classificação de Hata, análises de regressão logística univariada foram inicialmente conduzidas para investigar associações entre preditores basais e os dois desfechos compostos (ECAM e intervenção vascular). Posteriormente, foram construídos dois modelos de regressão logística multivariada para estimar *odds ratios* (OR) ajustadas e intervalo de confiança de 95% (IC 95%) da classificação de Hata, utilizando o Tipo V como categoria de referência.

As covariáveis incluídas nos modelos multivariados foram selecionadas segundo estratégia previamente definida. Idade ao diagnóstico e sexo foram incluídos *a priori* devido à sua reconhecida relevância clínica. Variáveis com associação univariada de p < 0,10 também foram incorporadas aos modelos finais, adotando-se um limiar mais inclusivo para reduzir a probabilidade de exclusão de preditores clinicamente relevantes durante o ajuste multivariado.

Todas as análises estatísticas foram realizadas no software R, versão 4.5.0. Nos modelos multivariados finais, foi adotado nível de significância estatística bilateral de p < 0,05.

## Resultados

### Características da coorte e dados basais

Um total de 203 pacientes com AT foi inicialmente identificado. Após a exclusão de 18 pacientes devido à classificação angiográfica incompleta ou ausência de informações de seguimento, 185 pacientes foram incluídos na análise final (Figura Central). A coorte foi estratificada de acordo com a classificação angiográfica de Hata, sendo o Tipo V o padrão predominante (64,9%), seguido pelos Tipos IV, IIa, IIb e III (Tabela 1).

**Tabela 1:** – Características demográficas e clínicas basais da coorte do estudo

Variável	Geral (n = 185)	Hata I (n = 16)	Hata IIa (n = 14)	Hata IIb (n = 10)	Hata III (n = 9)	Hata IV (n = 16)	Hata V (n = 120)	Valor de p
Idade ao diagnóstico, anos	29,0 (20,0-36,0)	30,0 (26,0-40,5)	29,0 (19,0-32,0)	29,0 (18,0-33,0)	23,0 23,0-45,0)	32,0 (19,5-39,0)	27,0 (20,0-35,5)	0,716
Intervalo entre início dos sintomas e diagnóstico, anos	1,0 (0,0-4,0)	1,0 (0,0-7,0)	0,0 (0,0-1,0)	2,0 (0,0-5,0)	1,0 (0,0-1,0)	2,5 (0,0-6,0)	1,0 (0,0-4,0)	0,561
Sexo feminino	148 (80,0)	15 (93,8)	11 (78,6)	9 (90,0)	7 (77,8)	10 (62,5)	96 (80,0)	0,369
Etnia branca	152 (82,2)	16 (100,0)	11 (78,6)	9 (90,0)	7 (77,8)	14 (87,5)	95 (79,2)	0,341
Etnia parda	12 (6,5)	0	0	1 (10,0)	1 (11,1)	1 (6,2)	9 (7,5)	0,672
Etnia negra	21 (11,3)	0	3 (21,4)	0	1 (11,1)	1 (6,2)	16 (13,3)	0,392
Hipertensão arterial sistêmica	138 (74,6)	9 (56,2)	9 (64,3)	7 (70,0)	9 (100,0)	14 (87,5)	90 (75,0)	0,133
Hipertensão renovascular	57 (30,8)	3 (18,8)	2 (14,3)	1 (10,0)	5 (55,6)	9 (56,2)	37 (30,8)	0,036
Insuficiência aórtica	40 (21,6)	2 (12,5)	0	1 (10,0)	2 (22,2)	1 (6,2)	34 (28,3)	0,047
Diabetes melito	29 (15,7)	4 (25,0)	4 (28,6)	2 (20,0)	1 (11,1)	2 (12,5)	16 (13,3)	0,509
Dislipidemia	108 (58,4)	8 (50,0)	7 (50,0)	4 (40,0)	4 (44,4)	9 (56,2)	76 (63,3)	0,497
Histórico de malignidade	6 (3,2)	0	1 (7,1)	0	0	2 (12,5)	3 (2,5)	0,253

*Os dados são apresentados como mediana (intervalo interquartil) ou n (%).*

A coorte total foi composta predominantemente por mulheres (80,0%) e indivíduos brancos (82,2%), com mediana de idade ao diagnóstico de 29,0 anos (IIQ, 20,0-36,0). Não foram observadas diferenças relevantes entre os grupos da classificação de Hata em relação à idade, distribuição por sexo ou tempo entre o início dos sintomas e o diagnóstico. Em contrapartida, as comorbidades basais variaram de acordo com o padrão angiográfico. A HRV foi observada com maior frequência entre pacientes classificados nos Tipos II-IV de Hata, enquanto insuficiência aórtica foi mais comum entre pacientes com doença Tipo V (Tabela 1).

### Seguimento, estado clínico e manejo terapêutico

As características do seguimento e os perfis terapêuticos estão apresentados na Tabela 2. A duração mediana do seguimento foi de 10,8 anos (IIQ, 4,5-15,8), com tempos de acompanhamento amplamente semelhantes entre os grupos da classificação de Hata. Na avaliação final, mais de 90% dos pacientes encontravam-se em remissão clínica (ITAS2010 < 2). Em relação à situação dos pacientes ao término do estudo, aproximadamente metade da coorte permanecia em acompanhamento ativo, enquanto proporções menores haviam recebido alta ou perdido o seguimento.

**Tabela 2:** – Características do seguimento, estado da doença e manejo terapêutico segundo a classificação de Hata

Variável	Geral (n = 185)	Hata I (n = 16)	Hata IIa (n = 14)	Hata IIb (n = 10)	Hata III (n = 9)	Hata IV (n = 16)	Hata V (n = 120)	Valor de p
**Estado da doença no último seguimento**								
Doença ativa	12 (6,5)	0	2 (14,3)	2 (20,0)	0	1 (6,2)	7 (5,8)	0,363
Remissão clínica	173 (93,5)	16 (100,0)	12 (85,7)	8 (80,0)	9 (100,0)	15 (93,8)	113 (94,2)	0,363
Duração do seguimento, anos	10,8 (4,5-15,8)	9,8 (4,3-14,2)	4,8 (3,0-13,8)	11,5 (4,5-15,1)	7,7 (3,4-15,7)	13,7 (7,7-16,9)	11,0 (5,0-15,9)	0,444
**Estratégia terapêutica inicial**								
Sem tratamento	33 (17,8)	4 (25,0)	4 (28,6)	1 (10,0)	2 (22,2)	3 (18,8)	19 (15,8)	0,702
Glicocorticoides	127 (68,6)	12 (75,0)	10 (71,4)	9 (90,0)	6 (66,7)	10 (62,5)	80 (66,7)	0,731
Metotrexato	133 (71,9)	11 (68,8)	9 (64,3)	8 (80,0)	7 (77,8)	13 (81,2)	85 (70,8)	0,918
Azatioprina	70 (37,8)	4 (25,0)	5 (35,7)	6 (60,0)	4 (44,4)	2 (12,5)	49 (40,8)	0,132
Micofenolato de mofetila	36 (19,5)	1 (6,2)	0	0	1 (11,1)	0	34 (28,3)	0,002
Leflunomida	38 (20,5)	4 (25,0)	3 (21,4)	4 (40,0)	1 (11,1)	3 (18,8)	23 (19,2)	0,680
Infliximabe	30 (16,2)	1 (6,2)	3 (21,4)	6 (60,0)	0	1 (6,2)	19 (15,8)	0,009
Tocilizumabe	13 (7,0)	0	0	3 (30,0)	0	0	10 (8,3)	0,101
**Regime terapêutico atual**								
Sem tratamento	116 (62,7)	10 (62,5)	11 (78,6)	6 (60,0)	7 (77,8)	12 (75,0)	70 (58,3)	0,532
Glicocorticoides	40 (21,6)	2 (12,5)	4 (28,6)	2 (20,0)	2 (22,2)	0	30 (25,0)	0,186
Metotrexato	26 (14,1)	1 (6,2)	1 (7,1)	2 (20,0)	1 (11,1)	0	21 (17,5)	0,375
Azatioprina	24 (13,0)	3 (18,8)	1 (7,1)	0	1 (11,1)	1 (6,2)	18 (15,0)	0,764
Micofenolato de mofetila	11 (5,9)	1 (6,2)	0	1 (10,0)	0	0	9 (7,5)	0,817
Leflunomida	11 (5,9)	1 (6,2)	2 (14,3)	1 (10,0)	0	2 (12,5)	5 (4,2)	0,240
Infliximabe	16 (8,6)	1 (6,2)	2 (14,3)	2 (20,0)	0	1 (6,2)	10 (8,3)	0,638
Tocilizumabe	5 (2,7)	0	0	2 (20,0)	0	0	3 (2,5)	0,161
Ácido acetilsalicílico	151 (81,6)	15 (93,8)	12 (85,7)	9 (90,0)	9 (100,0)	13 (81,2)	93 (77,5)	0,465
Estatinas	117 (63,2)	10 (62,5)	6 (42,9)	5 (50,0)	4 (44,4)	9 (56,2)	83 (69,2)	0,216

*Os dados são apresentados como mediana (intervalo interquartil) ou n (%).*

A análise das estratégias terapêuticas demonstrou diferenças significativas na prescrição de terapias imunomoduladoras específicas conforme o padrão angiográfico. Pacientes com Hata Tipo V receberam micofenolato de mofetila como terapia inicial com maior frequência, enquanto o uso de infliximabe foi mais comum entre pacientes com doença Tipo IIb. Em contraste, a utilização de glicocorticoides e metotrexato foi semelhante entre os subgrupos da classificação de Hata (Tabela 2).

### Incidência de desfechos em longo prazo e intervenções vasculares

A incidência de desfechos clínicos e procedimentos vasculares durante o seguimento está apresentada na Tabela 3. ECAM ocorreram em 35,7% dos pacientes, sendo predominantemente atribuídos a AVE (17,0%) e IC (15,7%). IAM (9,2%) e dissecção de aorta (3,2%) ocorreram com menor frequência. A incidência global de ECAM, bem como a incidência de seus componentes individuais, foi amplamente semelhante entre os padrões angiográficos de Hata, sem evidência de gradiente progressivo de risco conforme a distribuição anatômica da doença. Não foram identificadas diferenças significativas na ocorrência de ECAM (p = 0,261) nem em seus componentes individuais entre os grupos da classificação de Hata.

**Tabela 3:** – Incidência de desfechos clínicos e intervenções vasculares de acordo com a classificação angiográfica de Hata

Variável	Geral (n = 185)	Hata I (n = 16)	Hata IIa (n = 14)	Hata IIb (n = 10)	Hata III (n = 9)	Hata IV (n = 16)	Hata V (n = 120)	Valor de p
**ECAM**	66 (35,7)	5 (31,2)	3 (21,4)	2 (20,0)	1 (11,1)	8 (50,0)	47 (39,2)	0,261
AVE	31 (16,8)	4 (25,0)	2 (14,3)	1 (10,0)	0	3 (18,8)	21 (17,5)	0,765
IAM	17 (9,2)	0	2 (14,3)	2 (20,0)	1 (11,1)	2 (12,5)	10 (8,3)	0,372
IC	29 (15,7)	0	0	2 (20,0)	0	3 (18,8)	23 (19,2)	0,262
Dissecção de aorta	6 (3,2)	0	0	0	0	2 (12,5)	4 (3,3)	0,415
HP	12 (6,5)	0	1 (7,1)	1 (10,0)	0	0	10 (8,3)	0,727
TEV (TVP/EP)	16 (8,6)	0	0	1 (10,0)	0	3 (18,8)	12 (10,0)	0,341
Achados ecocardiográficos anormais*	85 (45,9)	5 (31,2)	5 (35,7)	5 (50,0)	1 (11,1)	5 (31,2)	64 (53,3)	0,061
**Intervenção vascular**	69 (37,3)	1 (6,2)	3 (21,4)	3 (30,0)	3 (33,3)	5 (31,2)	54 (45,0)	0,028
Implantação de endoprótese	34 (18,4)	1 (6,2)	1 (7,1)	1 (10,0)	2 (22,2)	4 (25,0)	25 (20,8)	0,571
Cirurgia de revascularização	14 (7,6)	0	0	1 (10,0)	2 (22,2)	1 (6,2)	10 (8,3)	0,343
Cirurgia valvar	14 (7,6)	0	0	1 (10,0)	0	1 (6,2)	12 (10,0)	0,691
Correção de aneurisma de aorta	8 (4,3)	0	2 (14,3)	0	0	0	6 (5,0)	0,475
Mortalidade por todas as causas	6 (3,2)	2 (12,5)	0	0	0	0	4 (3,3)	0,415

Os dados são apresentados como n (%). *Presença de alterações cardíacas estruturais ou funcionais, incluindo DSVE, DVC, DRA ou HP. AVE: acidente vascular encefálico; DRA: dilatação da raiz da aorta; DSVE: disfunção sistólica do ventrículo esquerdo; DVC: doença valvar cardíaca; ECAM: eventos cardiovasculares adversos maiores; EP: embolia pulmonar; HP: hipertensão pulmonar; IAM: infarto agudo do miocárdio; IC: insuficiência cardíaca; TEV: tromboembolismo venoso; TVP: trombose venosa profunda.

O desfecho composto de intervenção vascular ocorreu em 37,3% dos pacientes e incluiu implantação de endoprótese (18,0%), cirurgia de revascularização (7,6%), cirurgia valvar (7,6%) e correção de aneurisma de aorta (4,3%). Diferentemente dos ECAM, a necessidade de intervenção vascular variou conforme a classificação de Hata, com maior frequência de procedimentos observada em pacientes Tipo V e menor frequência em pacientes Tipo I. Entretanto, quando analisados individualmente, os procedimentos vasculares específicos não diferiram significativamente entre os subgrupos angiográficos.

### Análise prognóstica de fatores basais

As análises de regressão logística univariada, utilizando Hata Tipo V como categoria de referência, identificaram preditores basais de desfechos em longo prazo. Para ECAM (Tabela Suplementar 1), maior tempo entre o início dos sintomas e o diagnóstico e sexo masculino estiveram associados a maior risco, enquanto HRV apresentou associação limítrofe. A classificação angiográfica de Hata não demonstrou associação significativa com ECAM nas análises univariadas.

Em relação à intervenção vascular, Hata Tipo I associou-se a probabilidade substancialmente menor de intervenção quando comparado à doença Tipo V. Além disso, HAS e insuficiência aórtica estiveram associadas a maior risco de necessidade de procedimentos vasculares (Tabela Suplementar 2).

### Análise multivariada de fatores prognósticos

As análises de regressão logística multivariada, ajustadas de acordo com a estratégia de modelagem previamente definida (Tabela Suplementar 3), demonstraram que a classificação de Hata não predisse independentemente a ocorrência de ECAM. O sexo feminino permaneceu como fator protetor independente, enquanto maior intervalo entre o início dos sintomas e o diagnóstico persistiu como preditor independente de aumento de risco. A HRV perdeu significância estatística após o ajuste multivariado (Figura Central).

Em relação à intervenção vascular, Hata Tipo I permaneceu como forte fator protetor independente em comparação com doença Tipo V, enquanto insuficiência aórtica basal permaneceu independentemente associada a maior risco de procedimentos. A HAS apresentou apenas tendência não significativa de aumento de risco após o ajuste multivariado.

## Discussão

Neste estudo de coorte de centro único, com seguimento mediano superior a 1 década, avaliamos o valor prognóstico da classificação angiográfica de Hata para desfechos em longo prazo em 185 pacientes com AT. Os principais achados foram os seguintes: i) a classificação de Hata não se mostrou preditora independente de ECAM; ii) demonstrou ser um forte preditor independente da necessidade futura de intervenções vasculares; iii) o Hata Tipo I apresentou efeito protetor independente contra intervenções vasculares quando comparado ao padrão mais extenso Hata Tipo V; iv) insuficiência aórtica basal predisse independentemente a necessidade de intervenção vascular; e v) sexo feminino e menor atraso diagnóstico exerceram efeito protetor independente sobre a ocorrência de ECAM (Figura Central).

Nossos achados indicam que a classificação de Hata não prediz ECAM, desafiando a premissa de que essa estrutura anatômica amplamente utilizada se traduz diretamente em estratificação prognóstica cardiovascular. Essa observação é particularmente relevante considerando que o Tipo V foi o padrão angiográfico predominante em nossa coorte (64,9%), em concordância com evidências prévias oriundas de metanálises,^8^ além de previamente associado a maior atividade da doença.^13^ Uma possível explicação é que, embora a classificação de Hata identifique de forma eficiente a extensão do acometimento arterial, ela pode não capturar adequadamente os mecanismos envolvidos nas principais complicações isquêmicas. O risco de ECAM provavelmente depende mais fortemente de manifestações específicas de alto risco do que exclusivamente da distribuição anatômica da doença. Essa interpretação é sustentada por grandes estudos de coorte que identificaram determinantes prognósticos independentes da classificação angiográfica, incluindo curso progressivo da doença, acometimento da aorta torácica, retinopatia,^5,14^ atraso diagnóstico e insuficiência aórtica basal,^6^ fatores mais diretamente relacionados à evolução cardiovascular desfavorável.

Um achado importante de nosso modelo prognóstico foi o efeito protetor independente do sexo feminino sobre ECAM (OR, 0,38). Esse resultado está alinhado a evidências emergentes que demonstram diferenças fenotípicas relacionadas ao sexo na AT. Grandes coortes provenientes da China e do Japão demonstraram que pacientes do sexo masculino desenvolvem com maior frequência complicações graves, incluindo aneurismas de aorta, disfunção renal e HAS.^15,16^ De forma semelhante, um estudo multicêntrico francês identificou o sexo masculino como preditor independente de recaída.^5^ Embora alguns estudos tenham demonstrado achados divergentes, incluindo maior prevalência de regurgitação aórtica entre mulheres,^17^ a maior parte das evidências aponta para maior carga de manifestações graves entre homens, reforçando nosso achado de menor risco cardiovascular em mulheres.

Em contraste com os ECAM, nossos resultados demonstraram associação independente robusta entre a classificação de Hata e a necessidade de intervenções vasculares em longo prazo. A taxa global de intervenção de 37,3% observada em nossa coorte está em consonância com estudos longitudinais de grande porte que relatam taxas de revascularização variando entre 41,8% e 59%,^5,18,19^ destacando a contribuição substancial dos procedimentos vasculares para a carga da doença em longo prazo. A estratificação de risco segundo o padrão angiográfico foi particularmente evidente. A doença Tipo V apresentou a maior carga de intervenções (45,0%), enquanto o Tipo I apresentou a menor taxa (6,3%). A análise multivariada confirmou o Tipo I como forte fator protetor (OR, 0,10), independentemente de outras características basais. A insuficiência aórtica basal também se manteve como preditora independente de intervenção vascular (OR, 2,54).

Esses achados sugerem que o acometimento vascular difuso característico da doença Tipo V aumenta a necessidade de procedimentos por múltiplos mecanismos. Embora o Tipo V tenha sido associado a maior prevalência de insuficiência aórtica basal (28,3%), seu impacto prognóstico parece ir além do comprometimento valvar isolado, provavelmente refletindo a carga cumulativa de lesões estenóticas, oclusivas e aneurismáticas que demandam abordagem cirúrgica ou endovascular em múltiplos territórios vasculares. Em conjunto, essas observações sustentam a utilidade clínica da classificação de Hata como ferramenta prática baseada em métodos de imagem para estratificação de risco procedimental em longo prazo na AT. Pacientes classificados como Tipo V podem representar um fenótipo de maior risco, justificando monitoramento mais rigoroso e avaliação mais precoce pela cirurgia vascular, enquanto o Tipo I identifica um subgrupo comparativamente de menor risco.

Além da classificação anatômica, nossos achados também enfatizam a carga de dano vascular já estabelecida no momento do diagnóstico. HAS estava presente em 75% dos pacientes, enquanto aproximadamente um terço apresentava HRV. Essa elevada carga de lesão vascular provavelmente reflete o atraso diagnóstico frequentemente observado na AT, uma doença caracterizada por apresentação insidiosa e reconhecimento tardio.^5^ Nossa análise reforça ainda a relevância prognóstica do atraso diagnóstico, uma vez que maior tempo entre o início dos sintomas e o diagnóstico aumentou independentemente o risco de ECAM (OR, 1,10), em concordância com evidências prospectivas prévias que identificaram atrasos diagnósticos superiores a 3-4 anos como preditores independentes de ECAM e mortalidade.^6,20^

Outro achado relevante foi a dissociação entre remissão clínica e progressão vascular em longo prazo. Na avaliação final do seguimento, 92,4% dos pacientes foram classificados em remissão clínica (ITAS2010 < 2), porém as taxas cumulativas de intervenção vascular (37,3%) e ECAM (35,7%) permaneceram substanciais. Esses resultados sugerem que medidas convencionais de atividade da doença e biomarcadores inflamatórios sistêmicos podem refletir de forma incompleta o dano vascular persistente. Observações semelhantes já foram descritas anteriormente, incluindo evidências de arterite ativa em 44% de espécimes cirúrgicos obtidos de pacientes considerados clinicamente inativos.^4^

Esses achados reforçam a importância da avaliação vascular avançada para além da análise de sintomas e biomarcadores inflamatórios isoladamente. Na prática clínica, esses resultados sustentam estratégias estruturadas de acompanhamento baseadas em métodos de imagem, incorporando angiotomografia computadorizada seriada ou angiorressonância magnética e, em casos selecionados, tomografia por emissão de pósitrons/tomografia computadorizada, com o objetivo de aprimorar o monitoramento da progressão da doença e otimizar tanto o tratamento clínico quanto o momento ideal para intervenções de revascularização.

Também identificamos diferenças nas estratégias terapêuticas iniciais entre os grupos da classificação de Hata, particularmente maior utilização de micofenolato de mofetila entre pacientes com doença Tipo V e maior uso de infliximabe entre aqueles com doença Tipo IIb. Esses padrões terapêuticos provavelmente refletem viés de indicação, e não algoritmos terapêuticos fundamentados em evidência, uma vez que médicos assistentes podem optar preferencialmente por tratamentos mais intensivos em fenótipos percebidos como de maior risco vascular. Embora a efetividade terapêutica não tenha sido avaliada no presente estudo, esses resultados ilustram como fenótipos angiográficos podem influenciar a tomada de decisão terapêutica na prática clínica rotineira.

Este estudo apresenta algumas limitações. Seu delineamento retrospectivo introduz possibilidade de viés de informação, embora procedimentos padronizados de coleta de dados tenham sido adotados para minimizar essa limitação. Além disso, 24,3% dos pacientes perderam seguimento, o que pode ter introduzido viés de atrito. Entretanto, o longo período mediano de seguimento de 10,8 anos confere robustez à avaliação de desfechos em longo prazo. Finalmente, o desenho de centro único pode limitar a generalização dos achados para populações com diferentes características étnicas ou genéticas. Considerando essas limitações, especialmente o delineamento retrospectivo e o seguimento incompleto, nossos achados devem ser interpretados com cautela e predominantemente como geradores de hipótese. Estudos prospectivos multicêntricos são necessários para validar essas observações e esclarecer de forma mais robusta as relações causais entre os padrões angiográficos de Hata, a ocorrência de ECAM e a necessidade de intervenções vasculares em longo prazo na AT.

## Conclusão

Embora a classificação angiográfica de Hata não tenha demonstrado capacidade de predizer ECAM em longo prazo, ela emergiu como importante preditora independente da necessidade futura de intervenções vasculares na AT. O acometimento vascular extenso, particularmente na doença Hata Tipo V, associou-se a maior risco de procedimentos independentemente de outros fatores, incluindo insuficiência aórtica basal.

Adicionalmente, o impacto do atraso diagnóstico e a dissociação observada entre remissão clínica e progressão vascular silenciosa reforçam a importância de estratégias de monitoramento baseadas em métodos de imagem no manejo da AT. Esses achados também ressaltam a necessidade de estudos prospectivos multicêntricos para validar e refinar as implicações prognósticas dos padrões angiográficos na AT.

## Material suplementar

Tabelas suplementares
