# Supplementary material for: Valor Prognóstico da Classificação Angiográfica de Hata para Desfechos em Longo Prazo na Arterite de Takayasu: Um Estudo Retrospectivo de Centro Único
Source: Arq Bras Cardiol. 2026 Jun 15;123(6):e20250786. [Article in Portuguese] doi: 10.36660/abc.20250786 (PMC13400063; doi:10.36660/abc.20250786)
Supplement: Tabelas suplementares [file 0066-782x-abc-123-6-e20250786-suppl01.pdf]

Supplement Table 1. Univariate analysis for MACE

	n	OR	95%CI	*P* value
Angiographic classification				
Hata	185			
V		-	-	
I		0.71	0.21-2.07	0.557
IIa		0.42	0.09-1.44	0.220
IIb		0.39	0.06-1.63	0.264
III		0.19	0.01-1.11	0.167
IV		1.55	0.54-4.50	0.418
Age at diagnosis	185	1.02	0.99-1.05	0.187
Onset to diagnosis time	185	1.1	1.04-1.18	0.001
Sex (female)	185	0.44	0.21-0.92	0.028
Hypertension	185	1.63	0.80-3.46	0.191
Renovascular hypertension	185	1.85	0.97-3.51	0.061
Aortic insufficiency	185	1.65	0.81-3.37	0.169
Diabetes mellitus	185	1.58	0.70-3.52	0.167
Dyslipidemia	185	1.41	0.76-2.63	0.278
White	185	1.34	0.61-3.14	0.184
Parda	185	1.31	0.37-4.28	0.665
Black	185	0.53	0.17-1.42	0.241

CI: confidence interval; OR: odds ratio.

Supplement Table 2. Univariate analysis for vascular interventions

	n	OR	95%CI	*P* value
Angiographic classification				
Hata	185			
I		0.08	0.00-0.42	0.017
IIa		0.33	0.07-1.13	0.118
IIb		0.52	0.11-1.98	0.375
III		0.61	0.12-2.43	0.519
IV		0.56	0.17-1.63	0.315
Age at diagnosis	185	1.00	0.97-1.03	0.913
Onset to diagnosis time	185	1.04	0.99-1.09	0.151
Sex (female)	185	0.55	0.27-1.15	0.106
Hypertension	185	2.37	1.14-5.25	0.025
Renovascular hypertension	185	1.34	0.70-2.54	0.373
Aortic insufficiency	185	3.33	1.63-6.98	0.001
Diabetes mellitus	185	1.03	0.44-2.31	0.944
Dyslipidemia	185	0.97	0.53-1.79	0.922
White	185	1.05	0.49-2.35	0.903
Parda	185	1.22	0.35-3.97	0.748
Black	185	0.82	0.30-2.09	0.689

CI: confidence interval; OR: odds ratio.

Supplement Table 3. Multivariate analysis for MACE and vascular interventions

	**OR**	**95%CI**	***P* value**
MACE			
Angiographic classification			
Hata			
I	0.71	0.19-2.24	0.586
IIa	0.49	0.10-1.74	0.328
IIb	0.43	0.06-1.90	0.338
III	0.14	0.01-0.92	0.085
IV	1.01	0.31-3.19	0.987
Age at diagnosis	1.02	0.98-1.05	0.261
Sex (female)	0.38	0.17-0.86	0.021
Onset to diagnosis time	1.10	1.03-1.18	0.006
Renovascular hypertension	1.60	0.77-3.34	0.209
Vascular Intervention			
Hata			
I	0.10	0.01-0.53	0.029
IIa	0.46	0.10-1.61	0.273
IIb	0.66	0.13-2.62	0.588
III	0.52	0.10-2.17	0.405
IV	0.55	0.15-1.72	0.337
Age at diagnosis	1.00	0.98-1.03	0.801
Female	0.58	0.26-1.30	0.185
Aortic insufficiency	2.54	1.18-5.60	0.018
Hypertension	2.06	0.93, 4.80	0.081

CI: confidence interval; MACE: major adverse cardiovascular events; OR: odds ratio.
