# Supplementary material for: Valor Prognóstico da Classificação Angiográfica de Hata para Desfechos em Longo Prazo na Arterite de Takayasu: Um Estudo Retrospectivo de Centro Único
Source: Arq Bras Cardiol. 2026 Jun 15;123(6):e20250786. [Article in Portuguese] doi: 10.36660/abc.20250786 (PMC13400063; doi:10.36660/abc.20250786)
Supplement: Translation

# Translation: Prognostic Value of the Hata Angiographic Classification for Long-Term Outcomes in Takayasu Arteritis: A Single-Center, Retrospective Study

**DOI:** 10.36660/abc.20250786i

**Authors:** Anisio Uchoa Leite Santana, Samuel Katsuyuki Shinjo

**Affiliations:** 1 Brazil Division of Rheumatology, Faculdade de Medicina da Universidade de São Paulo (FMUSP), São Paulo, SP – Brazil

**Keywords:** Takayasu Arteritis, Prognosis, Systemic Vasculitis

## Abstract

**Background:**

The prognostic utility of the Hata angiographic classification for long-term outcomes in Takayasu arteritis (TAK) remains unclear.

**Objectives:**

To evaluate the prognostic value of the Hata classification for predicting major adverse cardiovascular events (MACE) and the need for vascular interventions in patients with TAK.

**Methods:**

This single-center, retrospective cohort study included patients diagnosed with TAK between 2000 and 2025. Multivariate logistic regression analyses were performed to identify independent predictors, with statistical significance defined as p < 0.05.

**Results:**

Of 203 patients initially identified, 18 were excluded. The final cohort included 185 patients, of whom 80.0% were women, with a median age at diagnosis of 29 years. Type V was the predominant angiographic pattern (64.9%). During a median follow-up of 10.8 years, MACE occurred in 35.7% of patients, while 37.3% required vascular intervention. The Hata classification was not associated with MACE occurrence. Independent predictors of MACE included female sex, which was protective (odds ratio [OR], 0.38; 95% CI, 0.17-0.86; p = 0.021), and longer symptom onset-to-diagnosis time, which increased risk (OR, 1.10; 95% CI, 1.03-1.18; p = 0.006). In contrast, the Hata classification independently predicted the need for vascular intervention. Hata Type I showed a strong protective association compared with Type V (OR, 0.10; 95% CI, 0.01-0.53; p = 0.029). Baseline aortic insufficiency was also independently associated with a higher likelihood of intervention (OR, 2.54; 95% CI, 1.18-5.60; p = 0.018) (Central Illustration).

**Conclusions:**

The Hata classification appears to be a robust predictor of vascular intervention but not MACE in TAK. Greater anatomical disease extension (Type V) and baseline aortic insufficiency were associated with increased procedural risk, whereas MACE risk was associated with diagnostic delay and male sex. The observed long-term dissociation between high rates of clinical remission (92.4%) and continued disease progression underscores the importance of imaging-based surveillance and highlights the need for prospective multicenter studies to validate these findings.

## Introduction

Takayasu arteritis (TAK) is a rare, chronic granulomatous vasculitis that predominantly affects large-caliber arteries, particularly the aorta and its major branches.^1-3^ The disease primarily occurs in young women, typically during the second and third decades of life. The underlying inflammatory process promotes arterial wall thickening and fibrosis, ultimately leading to stenotic, occlusive, or aneurysmal lesions.^4^ These vascular abnormalities are major determinants of morbidity and mortality and frequently result in severe ischemic complications, including stroke, acute myocardial infarction (AMI), renovascular hypertension (RVH), and heart failure (HF).^5,6^

Because of the marked heterogeneity in clinical presentation and disease progression, predicting long-term outcomes in TAK remains challenging.^5^ To standardize the anatomical characterization of arterial involvement, several angiographic classification systems have been developed. Among these, the classification proposed by Hata et al.^7^ is the most widely adopted, categorizing disease into six angiographic patterns (Types I, IIa, IIb, III, IV, and V) according to the distribution of vascular involvement. This classification has become a fundamental framework for characterizing and comparing patient populations in clinical studies.

Previous investigations have demonstrated associations between Hata classification patterns and specific clinical phenotypes. A recent meta-analysis identified Type V, the most extensive angiographic subtype, as both the most prevalent pattern and the subtype associated with the highest overall symptom burden.^8^ Other studies have linked specific angiographic patterns to distinct complications, including an increased risk of cerebrovascular events in patients with Type IIa disease.^9^ Nevertheless, the prognostic profile of TAK remains complex. Large cohort studies have identified multiple independent predictors of adverse outcomes, including progressive disease course, thoracic aortic involvement, retinopathy, and increased inflammatory biomarkers.^5,6^ However, the prognostic value of the Hata angiographic classification itself remains incompletely understood.

Despite its widespread use for anatomical characterization, a critical knowledge gap persists regarding the direct prognostic implications of the Hata angiographic classification for long-term outcomes in TAK. Therefore, this study aimed to evaluate the prognostic value of the Hata classification by examining associations between angiographic patterns and long-term clinical outcomes in a large single-center cohort of patients with TAK. Additionally, we sought to investigate whether specific Hata subtypes contribute to risk stratification for major adverse cardiovascular events (MACE) and vascular interventions.

## Methods

### Study design

This retrospective single-center cohort study included patients diagnosed with TAK who were followed at the vasculitis unit of our tertiary care center between January 2000 and October 2025. The study was conducted in accordance with the principles of the Declaration of Helsinki and was approved by the institutional Human Research Ethics Committee.

Eligible participants were required to have a confirmed diagnosis of TAK according to both the American College of Rheumatology (ACR) 1990 classification criteria^10^ and the European Alliance of Associations for Rheumatology/ACR 2022 classification criteria.^11^ Patients with incomplete or unavailable angiographic classification data were excluded.

The study was reported in accordance with the STrengthening the Reporting of OBservational studies in Epidemiology guidelines.

### Data coll ection

Data were systematically extracted from electronic medical records using a standardized protocol. Variables included demographic and baseline characteristics, such as age at diagnosis, sex, ethnicity, and follow-up duration; angiographic classification according to Hata et al.^7^ (Types I, IIa, IIb, III, IV, and V); and treatment-related variables, including medications administered during follow-up, such as glucocorticoids, immunosuppressive agents, immunomodulatory agents, biologic therapies, antiplatelet agents, and statins.

Long-term outcomes and complications were also assessed, including mortality, MACE (AMI, HF, cerebrovascular events, and aortic dissection), and the need for vascular interventions. Additionally, comorbidities and clinical complications, including hypertension, RVH, aortic insufficiency, diabetes mellitus, dyslipidemia, and pulmonary hypertension, were recorded.

Two primary composite prognostic endpoints were defined: i) MACE, comprising stroke, AMI, HF, and aortic dissection; and ii) vascular intervention, defined as the requirement for endoprosthesis implantation, bypass surgery, valve surgery, or aortic aneurysm repair.

Disease status at the last follow-up visit was categorized as either active disease or remission. Disease activity was assessed using the Indian Takayasu Clinical Activity Score (ITAS2010),^12^ with scores ≥ 2 indicating active disease.

### Statistical analysis

Descriptive statistics were used to characterize the study cohort. Continuous variables did not follow a normal distribution according to the Shapiro-Wilk test and were therefore presented as median and interquartile range (IQR, 25th-75th percentile). Categorical variables were reported as frequencies and percentages.

Baseline characteristics across Hata classification groups were compared using the Kruskal-Wallis test for continuous variables and the chi-square test or Fisher’s exact test for categorical variables, without post hoc pairwise analyses.

To evaluate the prognostic value of the Hata classification, univariate logistic regression analyses were initially performed to assess associations between baseline predictors and the two composite outcomes (MACE and vascular intervention). Subsequently, two multivariable logistic regression models were constructed to estimate adjusted odds ratios (ORs) and 95% CIs for the Hata classification, using Type V as the reference category.

Covariates included in the multivariable models were selected according to a predefined strategy. Age at diagnosis and sex were included a priori because of their established clinical relevance. Variables with a univariate association of p < 0.10 were also incorporated into the final models, adopting a more inclusive threshold to reduce the likelihood of excluding clinically relevant predictors during multivariable adjustment.

All statistical analyses were performed using R version 4.5.0. Statistical significance in the final multivariable models was defined as a two-sided p-value < 0.05.

## Results

### Cohort characteristics and baseline data

A total of 203 patients with TAK were initially identified. After excluding 18 patients because of incomplete angiographic classification or missing follow-up information, 185 patients were included in the final analysis (Central Illustration). The cohort was stratified according to the Hata angiographic classification, with Type V representing the predominant pattern (64.9%), followed by Types IV, IIa, IIb, and III (Table 1).

**Table 1:** – Baseline demographic and clinical characteristics of the study cohort

Variable	Overall (n = 185)	Hata I (n = 16)	Hata IIa (n = 14)	Hata IIb (n = 10)	Hata III (n = 9)	Hata IV (n = 16)	Hata V (n = 120)	p-value
Age at diagnosis, years	29.0 (20.0-36.0)	30.0 (26.0-40.5)	29.0 (19.0-32.0)	29.0 (18.0-33.0)	23.0 23.0-45.0)	32.0 (19.5-39.0)	27.0 (20.0-35.5)	0.716
Symptom onset-to-diagnosis interval, years	1.0 (0.0-4.0)	1.0 (0.0-7.0)	0.0 (0.0-1.0)	2.0 (0.0-5.0)	1.0 (0.0-1.0)	2.5 (0.0-6.0)	1.0 (0.0-4.0)	0.561
Female sex	148 (80.0)	15 (93.8)	11 (78.6)	9 (90.0)	7 (77.8)	10 (62.5)	96 (80.0)	0.369
White ethnicity	152 (82.2)	16 (100.0)	11 (78.6)	9 (90.0)	7 (77.8)	14 (87.5)	95 (79.2)	0.341
Mixed race ethnicity	12 (6.5)	0	0	1 (10.0)	1 (11.1)	1 (6.2)	9 (7.5)	0.672
Black ethnicity	21 (11.3)	0	3 (21.4)	0	1 (11.1)	1 (6.2)	16 (13.3)	0.392
Hypertension	138 (74.6)	9 (56.2)	9 (64.3)	7 (70.0)	9 (100.0)	14 (87.5)	90 (75.0)	0.133
Renovascular hypertension	57 (30.8)	3 (18.8)	2 (14.3)	1 (10.0)	5 (55.6)	9 (56.2)	37 (30.8)	0.036
Aortic insufficiency	40 (21.6)	2 (12.5)	0	1 (10.0)	2 (22.2)	1 (6.2)	34 (28.3)	0.047
Diabetes mellitus	29 (15.7)	4 (25.0)	4 (28.6)	2 (20.0)	1 (11.1)	2 (12.5)	16 (13.3)	0.509
Dyslipidemia	108 (58.4)	8 (50.0)	7 (50.0)	4 (40.0)	4 (44.4)	9 (56.2)	76 (63.3)	0.497
History of malignancy	6 (3.2)	0	1 (7.1)	0	0	2 (12.5)	3 (2.5)	0.253

*Data are presented as median (interquartile range) or n (%).*

The overall cohort was predominantly female (80.0%) and White (82.2%), with a median age at diagnosis of 29.0 years (IQR, 20.0-36.0). No relevant differences were observed across Hata groups regarding age, sex distribution, or time from symptom onset to diagnosis. In contrast, baseline comorbidities varied according to angiographic pattern. RVH was more frequently observed among patients with Hata Types II-IV, whereas aortic insufficiency was more common in patients with Type V disease (Table 1).

Follow-up, clinical status, and therapeutic management

Patient follow-up characteristics and treatment profiles are summarized in Table 2. The median follow-up duration was 10.8 years (IQR, 4.5-15.8), with broadly similar follow-up times across Hata groups. At the final evaluation, more than 90% of patients were in clinical remission (ITAS2010 < 2). Regarding patient status at study completion, approximately half of the cohort remained under active follow-up, whereas smaller proportions had either been discharged or were lost to follow-up.

**Table 2:** – Follow-up characteristics, disease status, and therapeutic management by Hata classification

Variable	Overall (n = 185)	Hata I (n = 16)	Hata IIa (n = 14)	Hata IIb (n = 10)	Hata III (n = 9)	Hata IV (n = 16)	Hata V (n = 120)	p-value
**Disease status at last follow-up**								
Active disease status	12 (6.5)	0	2 (14.3)	2 (20.0)	0	1 (6.2)	7 (5.8)	0.363
Clinical remission	173 (93.5)	16 (100.0)	12 (85.7)	8 (80.0)	9 (100.0)	15 (93.8)	113 (94.2)	0.363
Follow-up duration, years	10.8 (4.5-15.8)	9.8 (4.3-14.2)	4.8 (3.0-13.8)	11.5 (4.5-15.1)	7.7 (3.4-15.7)	13.7 (7.7-16.9)	11.0 (5.0-15.9)	0.444
**Initial therapeutic strategy**								
No treatment	33 (17.8)	4 (25.0)	4 (28.6)	1 (10.0)	2 (22.2)	3 (18.8)	19 (15.8)	0.702
Glucocorticoids	127 (68.6)	12 (75.0)	10 (71.4)	9 (90.0)	6 (66.7)	10 (62.5)	80 (66.7)	0.731
Methotrexate	133 (71.9)	11 (68.8)	9 (64.3)	8 (80.0)	7 (77.8)	13 (81.2)	85 (70.8)	0.918
Azathioprine	70 (37.8)	4 (25.0)	5 (35.7)	6 (60.0)	4 (44.4)	2 (12.5)	49 (40.8)	0.132
Mycophenolate mofetil	36 (19.5)	1 (6.2)	0	0	1 (11.1)	0	34 (28.3)	0.002
Leflunomide	38 (20.5)	4 (25.0)	3 (21.4)	4 (40.0)	1 (11.1)	3 (18.8)	23 (19.2)	0.680
Infliximab	30 (16.2)	1 (6.2)	3 (21.4)	6 (60.0)	0	1 (6.2)	19 (15.8)	0.009
Tocilizumab	13 (7.0)	0	0	3 (30.0)	0	0	10 (8.3)	0.101
**Current therapeutic regimen**								
No treatment	116 (62.7)	10 (62.5)	11 (78.6)	6 (60.0)	7 (77.8)	12 (75.0)	70 (58.3)	0.532
Glucocorticoids	40 (21.6)	2 (12.5)	4 (28.6)	2 (20.0)	2 (22.2)	0	30 (25.0)	0.186
Methotrexate	26 (14.1)	1 (6.2)	1 (7.1)	2 (20.0)	1 (11.1)	0	21 (17.5)	0.375
Azathioprine	24 (13.0)	3 (18.8)	1 (7.1)	0	1 (11.1)	1 (6.2)	18 (15.0)	0.764
Mycophenolate mofetil	11 (5.9)	1 (6.2)	0	1 (10.0)	0	0	9 (7.5)	0.817
Leflunomide	11 (5.9)	1 (6.2)	2 (14.3)	1 (10.0)	0	2 (12.5)	5 (4.2)	0.240
Infliximab	16 (8.6)	1 (6.2)	2 (14.3)	2 (20.0)	0	1 (6.2)	10 (8.3)	0.638
Tocilizumab	5 (2.7)	0	0	2 (20.0)	0	0	3 (2.5)	0.161
Aspirin	151 (81.6)	15 (93.8)	12 (85.7)	9 (90.0)	9 (100.0)	13 (81.2)	93 (77.5)	0.465
Statins	117 (63.2)	10 (62.5)	6 (42.9)	5 (50.0)	4 (44.4)	9 (56.2)	83 (69.2)	0.216

*Data are presented as median (interquartile range) or n (%).*

Analysis of treatment strategies demonstrated significant differences in the prescription of selected immunomodulatory therapies according to angiographic pattern. Patients with Hata Type V more frequently received mycophenolate mofetil as initial therapy, whereas infliximab use was more common among patients with Type IIb disease. In contrast, glucocorticoid and methotrexate use was comparable across Hata subgroups (Table 2).

### Incidence of long-term outcomes and vascular interventions

The incidence of clinical outcomes and vascular procedures during follow-up is presented in Table 3. MACE occurred in 35.7% of patients and were primarily driven by stroke (17.0%) and HF (15.7%). AMI (9.2%) and aortic dissection (3.2%) occurred less frequently. Overall MACE incidence, as well as the incidence of its individual components, was broadly similar across Hata angiographic patterns, without evidence of a progressive risk gradient according to disease distribution. No significant differences were identified in MACE occurrence (p = 0.261) or in its individual components across Hata groups.

**Table 3:** – Incidence of clinical outcomes and vascular interventions according to Hata angiographic classification

Variable	Overall (n = 185)	Hata I (n = 16)	Hata IIa (n = 14)	Hata IIb (n = 10)	Hata III (n = 9)	Hata IV (n = 16)	Hata V (n = 120)	p-value
**MACE**	66 (35.7)	5 (31.2)	3 (21.4)	2 (20.0)	1 (11.1)	8 (50.0)	47 (39.2)	0.261
Stroke	31 (16.8)	4 (25.0)	2 (14.3)	1 (10.0)	0	3 (18.8)	21 (17.5)	0.765
AMI	17 (9.2)	0	2 (14.3)	2 (20.0)	1 (11.1)	2 (12.5)	10 (8.3)	0.372
HF	29 (15.7)	0	0	2 (20.0)	0	3 (18.8)	23 (19.2)	0.262
Aortic dissection	6 (3.2)	0	0	0	0	2 (12.5)	4 (3.3)	0.415
PH	12 (6.5)	0	1 (7.1)	1 (10.0)	0	0	10 (8.3)	0.727
VTE (DVT/PE)	16 (8.6)	0	0	1 (10.0)	0	3 (18.8)	12 (10.0)	0.341
Abnormal echocardiographic findings*	85 (45.9)	5 (31.2)	5 (35.7)	5 (50.0)	1 (11.1)	5 (31.2)	64 (53.3)	0.061
**Vascular intervention**	69 (37.3)	1 (6.2)	3 (21.4)	3 (30.0)	3 (33.3)	5 (31.2)	54 (45.0)	0.028
Endoprosthesis implantation	34 (18.4)	1 (6.2)	1 (7.1)	1 (10.0)	2 (22.2)	4 (25.0)	25 (20.8)	0.571
Bypass surgery	14 (7.6)	0	0	1 (10.0)	2 (22.2)	1 (6.2)	10 (8.3)	0.343
Valve surgery	14 (7.6)	0	0	1 (10.0)	0	1 (6.2)	12 (10.0)	0.691
Aortic aneurysm repair	8 (4.3)	0	2 (14.3)	0	0	0	6 (5.0)	0.475
All-cause mortality	6 (3.2)	2 (12.5)	0	0	0	0	4 (3.3)	0.415

Data are presented as n (%). *Presence of structural or functional cardiac abnormalities, including LVSD, VHD, ARD, or PH. AMI: acute myocardial infarction; ARD: aortic root dilation; DVT: deep vein thrombosis; HF: heart failure; LVSD: left ventricular systolic dysfunction; MACE: major adverse cardiovascular events; PE: pulmonary embolism; PH: pulmonary hypertension; VHD: valvular heart disease; VTE: venous thromboembolism.

The composite outcome of vascular intervention occurred in 37.3% of patients and included endoprosthesis implantation (18.0%), bypass surgery (7.6%), valve surgery (7.6%), and aortic aneurysm repair (4.3%). Unlike MACE, the requirement for vascular intervention varied according to Hata classification, with the highest procedural burden observed in Type V and the lowest in Type I disease. However, when analyzed individually, specific vascular procedures did not differ significantly across angiographic subgroups.

### Prognostic analysis of baseline factors

Univariate logistic regression analyses, using Hata Type V as the reference category, identified baseline predictors of long-term outcomes. For MACE (Supplementary Table 1), longer time from symptom onset to diagnosis and male sex were associated with higher risk, whereas RVH demonstrated a borderline association. Hata angiographic classification showed no significant association with MACE in univariate analyses.

Regarding vascular intervention, Hata Type I was associated with a substantially lower probability of intervention compared with Type V disease. Additionally, both hypertension and aortic insufficiency were associated with increased risk of requiring vascular procedures (Supplementary Table 2).

### Multivariate analysis of prognostic factors

Multivariable logistic regression analyses adjusted according to the predefined modeling strategy (Supplementary Table 3) demonstrated that Hata classification did not independently predict MACE occurrence. Female sex remained an independent protective factor, whereas longer onset-to-diagnosis time persisted as an independent predictor of increased risk. RVH lost statistical significance after multivariable adjustment (Central Illustration).

For vascular intervention, Hata Type I remained a strong independent protective factor compared with Type V disease, whereas baseline aortic insufficiency remained independently associated with increased procedural risk. Hypertension showed only a nonsignificant trend toward increased risk after multivariable adjustment.

## Discussion

In this single-center cohort study with a median follow-up exceeding 1 decade, we evaluated the prognostic value of the Hata angiographic classification for long-term outcomes in 185 patients with TAK. The main findings were as follows: i) the Hata classification was not an independent predictor of MACE; ii) it was a strong independent predictor of future vascular intervention requirements; iii) Hata Type I was independently protective against vascular intervention compared with the more extensive Hata Type V pattern; iv) baseline aortic insufficiency independently predicted vascular intervention; and v) female sex and shorter diagnostic delay independently protected against MACE occurrence (Central Illustration).

Our findings indicate that the Hata classification does not predict MACE, challenging the assumption that this widely adopted anatomical framework directly translates into prognostic cardiovascular stratification. This observation is particularly relevant because Type V was the predominant angiographic pattern in our cohort (64.9%), consistent with previous evidence from meta-analysis,^8^ and has previously been associated with greater disease activity.^13^ One possible explanation is that although the Hata classification effectively identifies the extent of arterial involvement, it may not adequately capture the mechanisms underlying major ischemic complications. MACE risk likely depends more strongly on specific high-risk disease manifestations than on anatomical distribution alone. This interpretation is supported by large cohort studies identifying prognostic determinants independent of angiographic classification, including progressive disease course, thoracic aortic involvement, retinopathy,^5,14^ diagnostic delay, and baseline aortic insufficiency,^6^ all factors more directly related to adverse cardiovascular evolution.

An important finding of our prognostic model was the independent protective effect of female sex against MACE (OR, 0.38). This result aligns with emerging evidence demonstrating sex-related phenotypic differences in TAK. Large cohorts from China and Japan have shown that male patients more frequently develop severe complications, including aortic aneurysms, kidney dysfunction, and hypertension.^15,16^ Similarly, a multicenter French study identified male sex as an independent predictor of relapse.^5^ Although some reports have demonstrated conflicting findings, including a higher prevalence of aortic regurgitation among women,^17^ most research supports a greater burden of severe disease manifestations among males, which reinforces our finding of lower cardiovascular risk in women.

In contrast to MACE, our results demonstrate a robust independent association between Hata classification and long-term vascular intervention requirements. The overall intervention rate of 37.3% observed in our cohort is consistent with major longitudinal studies reporting revascularization rates ranging from 41.8% to 59%,^5,18,19^ which highlights the substantial contribution of vascular procedures to long-term disease burden. Risk stratification according to angiographic pattern was particularly evident. Type V disease exhibited the highest intervention burden (45.0%), whereas Type I demonstrated the lowest rate (6.3%). Multivariable analysis confirmed Type I as a strong protective factor (OR, 0.10), independent of other baseline characteristics. Baseline aortic insufficiency also independently predicted vascular intervention (OR, 2.54).

These findings suggest that the diffuse vascular involvement characteristic of Type V disease increases procedural requirements through multiple mechanisms. Although Type V was associated with a higher prevalence of baseline aortic insufficiency (28.3%), its prognostic impact appears to extend beyond valvular disease alone, likely reflecting the cumulative burden of stenotic, occlusive, and aneurysmal lesions requiring surgical or endovascular management across multiple vascular territories. Collectively, these observations support the clinical utility of the Hata classification as a practical imaging-based tool for long-term procedural risk stratification in TAK. Patients classified as Type V may represent a higher-risk phenotype warranting closer surveillance and earlier vascular surgery assessment, whereas Type I identifies a comparatively lower-risk subgroup.

Beyond anatomical classification, our findings also emphasize the burden of established vascular damage at diagnosis. Hypertension was present in 75% of patients, whereas nearly one-third exhibited RVH. This substantial burden of vascular injury likely reflects the prolonged diagnostic delay frequently observed in TAK, a disease characterized by insidious presentation and delayed recognition.^5^ Our analysis further confirms the prognostic relevance of delayed diagnosis, as longer time from symptom onset to diagnosis independently increased risk of MACE (OR, 1.10), consistent with previous prospective evidence identifying diagnostic delays exceeding 3-4 years as independent predictors of MACE and mortality.^6,20^

Another noteworthy finding was the dissociation between clinical remission and long-term vascular progression. At the final follow-up evaluation, 92.4% of patients were classified as being in clinical remission (ITAS2010 < 2), yet cumulative rates of vascular intervention (37.3%) and MACE (35.7%) remained substantial. These findings suggest that conventional activity measures and systemic inflammatory markers may incompletely reflect persistent vascular injury. Similar observations have previously been reported, including evidence of active arteritis in 44% of surgical specimens obtained from patients considered clinically inactive.^4^

These observations reinforce the importance of advanced vascular imaging beyond symptom assessment and inflammatory biomarkers alone. In clinical practice, this may support structured imaging-based follow-up strategies incorporating serial computed tomography angiography or magnetic resonance angiography, complemented in selected cases by positron emission tomography/computed tomography, to improve monitoring of disease progression and optimize both medical treatment and revascularization timing.

We also identified differences in initial therapeutic strategies across Hata groups, particularly greater use of mycophenolate mofetil among patients with Type V disease and increased infliximab use among those with Type IIb disease. These treatment patterns likely reflect indication bias rather than evidence-based treatment algorithms, as clinicians may preferentially prescribe more intensive therapies for phenotypes perceived as carrying greater vascular risk. Although treatment effectiveness was not evaluated in the present study, these findings illustrate how angiographic phenotypes may influence therapeutic decision-making in routine clinical practice.

This study has several limitations. Its retrospective design introduces the possibility of information bias, although standardized data collection procedures were adopted to minimize this limitation. In addition, 24.3% of patients were lost to follow-up, which may have introduced attrition bias. Nevertheless, the long median follow-up duration of 10.8 years provides robust long-term outcome assessment. Finally, the single-center design may limit generalizability to populations with distinct ethnic or genetic backgrounds. Because of such limitations, particularly the retrospective design and incomplete follow-up, our findings should be interpreted cautiously and primarily as hypothesis-generating. Future prospective multicenter studies are warranted to validate these observations and further clarify the causal relationships between Hata angiographic patterns, MACE occurrence, and long-term vascular intervention requirements in TAK.

## Conclusion

Although the Hata angiographic classification did not predict long-term MACE, it emerged as an important independent predictor of future vascular intervention requirements in TAK. Extensive vascular involvement, particularly in Hata Type V disease, was associated with increased procedural risk independent of other factors, including baseline aortic insufficiency.

Additionally, the impact of diagnostic delay and the observed dissociation between clinical remission and ongoing silent vascular progression emphasize the importance of imaging-based surveillance strategies in management of TAK. These findings also reinforce the need for prospective multicenter studies to validate and further refine the prognostic implications of angiographic patterns in TAK.

## Supplemental Materials

Supplementary Tables
